# The Validation of the Perinatal Post-Traumatic Questionnaire in the Italian Population: Risk and Protective Factors

**DOI:** 10.3390/jcm14030704

**Published:** 2025-01-22

**Authors:** Odette Nardozza, Ilenia Passaquindici, Melba Emilia Persico, Antea D’Andrea, Chiara Suttora, Mirco Fasolo, Maria Spinelli

**Affiliations:** 1 Department of Psychology, G. D’Annunzio University of Chieti-Pescara, 66100 Chieti, Italy; odette.nardozza@phd.unich.it (O.N.); melbaemilia.persico@phd.unich.it (M.E.P.); mirco.fasolo@unich.it (M.F.); 2 Department of Neurosciences, Imaging and Clinical Sciences, G. D’Annunzio University of Chieti-Pescara, 66100 Chieti, Italy; passaquindici.ilenia@unich.it (I.P.); antea.dandrea@unich.it (A.D.); 3 Department of Psychology, University of Bologna, 40126 Bologna, Italy; chiara.suttora@unibo.it

**Keywords:** peripartum women mental health, peripartum disorders, women’s mental health, Perinatal Post-Traumatic Stress Disorder, psychometric properties

## Abstract

**Background:** Postpartum Post-Traumatic Stress Disorder (P-PTSD) symptoms develop after experiencing childbirth as traumatic. Several individual and environmental factors influence the childbirth experience. However, in Italy, this phenomenon remains unexplored due to the lack of screening tools. This project aims to validate the Italian version of the Perinatal PTSD Questionnaire (PPQ-II) and to identify maternal, pregnancy, childbirth, and postpartum risk and protective factors associated with P-PTSD. **Methods:** A total of 702 women (6–24 months postpartum) participated in an online cross-sectional study. They completed the PPQ-II, Depression Anxiety Stress Scales-21 (DASS-21), Big Five Inventory-10 (BFI-10), and a questionnaire to assess sociodemographic and perinatal factors. Factor structure was estimated with an exploratory (EFA) and confirmatory factor analysis (CFA). Internal consistency of the scale, convergent and divergent analyses were computed. Associations between perinatal factors and P-PTSD were also investigated. **Results:** The EFA revealed a two-factor structure: “Arousal and Mood alteration” and “Avoidance and Intrusion”. CFA supported the factor structure, showing a good fit of the data. The validity was confirmed by a significant association between the PPQ-II and the DASS-21 and a lower correlation with the BFI-10. Significant associations were found between P-PTSD symptoms and factors across the maternal, pregnancy, childbirth, and postpartum periods. **Conclusions:** The Italian PPQ-II is a valid screening tool to include in maternity care protocols for the early identification of P-PTSD. This study also contributes to identifying perinatal factors for symptom detection and the promotion of maternal well-being.

## 1. Introduction

Having a child is a major event of a woman’s life, characterised by significant physical, psychological, and social changes that define the development of maternal identity [1]. While becoming a mother is commonly considered a positive event, some women may experience childbirth as a traumatic experience [2]. This can occur when childbirth involves a real or perceived threat to the life or physical integrity of the mother and/or child, eliciting intense emotional reactions of stress and anxiety in the woman [3,4].

In certain cases, experiencing traumatic childbirth leads to the development of symptoms similar to those typical of Post Traumatic Stress Disorder (PTSD) [5]. According to the DSM-5, PTSD includes the following symptoms: intrusions of trauma-related memories that lead to re-experiencing the traumatic event (e.g., flashbacks, nightmares, or dissociative reactions); avoidance behaviours and thoughts related to trauma reminders (e.g., places, people, or feelings); changes in mood and cognition; and hyperarousal due to an increased state of physiological alertness to distress (e.g., irritability, difficulty sleeping, or being easily startled) [6]. When PTSD occurs after childbirth, it is commonly referred to as Perinatal Post-Traumatic Stress Disorder (P-PTSD) [7]. The stress and anxiety associated with a traumatic birth experience can manifest through nightmares and flashbacks related to the childbirth, as well as avoidance behaviours triggered by stimuli associated with the event, such as hospitals, doctors and, in severe cases, one’s own child [8]. Additionally, women with P-PTSD may experience negative emotions when exposed to trauma-related cues, persistent feelings of inadequacy as a mother, and emotional disconnection from their partner and child. Other possible symptoms include sleep disturbances, difficulty concentrating, or hypervigilance as a means to prevent future traumatic experiences [9]. It is estimated that approximately 3–6% of the global female population develops symptoms of P-PTSD after childbirth, ranging from 4% in England to 12% in Turkey [5,10,11]. In high-risk groups, such as women with physical and psychological complications during pregnancy and postpartum (e.g., major depression, anxiety, gestational diabetes, etc.), the prevalence of P-PTSD symptoms increases to 15.7% [4]. The present study aimed to validate the Italian version of a questionnaire for assessing P-PTSD and to explore risk and protective factors associated with the phenomenon.

### 1.1. P-PTSD Short- and Long-Term Outcomes

The negative effects of P-PTSD on women’s wellbeing are varied, highlighting the need to address this phenomenon from both research and clinical perspectives. For instance, avoiding hospitals and healthcare professionals, which can trigger memories of the traumatic childbirth experience, may lead women to avoid medical visits altogether. This not only raises the risk of developing physical health conditions, but also increases the likelihood of anxiety, depression, and exacerbation of P-PTSD symptoms, due to the intense pain of recalling the traumatic event [12,13]. An English study confirmed that symptoms of P-PTSD are also related to fear of future childbirth, which can lead women to make difficult choices not to have more children [14]. Additionally, the cognitive changes resulting from traumatic childbirth may lead women to perceive others as potential threats, negatively impacting the quality of their social relationships, including those with their partner and child [15,16]. In another English study, it is estimated that couples experiencing P-PTSD, 7 to 18 months postpartum, report significant negative changes in their physical and psychological well-being, sexual intimacy, communication as a couple, feelings of blame related to the birth, and fears regarding future pregnancies [17]. Consequently, the psychological distress experienced by mothers due to P-PTSD and the decline in partner relationship quality may impair their ability to recognize and respond appropriately to their child’s needs, creating a negative cascading effect on the quality of the mother–infant emotional bond, as well as on the child’s subsequent cognitive and socio-emotional development [18,19]. It is possible to hypothesize the formation of a vicious cycle, where the symptoms of P-PTSD adversely affect the woman’s social functioning, while the child’s emotional and behavioural dysregulation exacerbates the mother’s psychological distress. Untreated P-PTSD symptoms also have a significant economic cost, with an impact of GBP 8.1 billion in the UK and USD 14 billion in the US for each annual birth cohort [11]. Despite these high costs, a study conducted in 18 European countries revealed serious gaps in the formalization of protocols for the assessment and treatment of psychological trauma at birth. To address this, it is essential that policymakers adopt national guidelines [20].

### 1.2. Risk and Protective Factors of P-PTSD

Individual and environmental factors for the development of P-PTSD have been studied to identify and intervene early in the emergence of symptoms [21]. These risk and protective factors can be grouped into those preceding the pregnancy, those related to the pregnancy itself, factors associated with childbirth, and those emerging during the post-partum period.

Individual vulnerabilities in a woman’s life history can be significant pre-pregnancy risk factors. Women who have experienced trauma, such as sexual abuse, emotional neglect in childhood, and/or being a victim or witness of domestic violence, are at higher risk to re-experiencing trauma during childbirth [9,22]. Having a family history of mood and personality disorders (i.e., depression, borderline personality disorder, narcissistic personality disorder, etc.) is an additional vulnerability that increases the risk of developing P-PTSD symptoms [23]. Furthermore, a low socioeconomic status (SES) is associated with elevated P-PTSD symptoms, primarily because it limits women’s access to adequate healthcare, increasing the risk of medical complications during childbirth and the puerperium [24]. A history of prior prenatal losses is another important risk factor for developing P-PTSD symptoms; indeed, women with multiple previous losses (including miscarriage, elective abortion, or stillbirth) are more likely to develop P-PTSD symptoms than those with no prior losses or only one loss [25].

Pregnancy-related risk factors include maternal behaviours often associated with the onset of obstetric complications, such as smoking, alcohol, and drug use [26]. Additionally, economic difficulties, relationship conflicts, or being single are conditions that make daily life more challenging, leading to increased stress during pregnancy [27].

Regarding factors associated with childbirth, the duration of labour, the type of delivery, and the pain perceived during childbirth are the main risk factors for the development of P-PTSD symptoms. Specifically, a longer and more painful labour and delivery contribute to a negative perception of the childbirth experience. Anesthesia may serve as a protective factor, helping to alleviate both physical pain and psychological distress; however, certain adverse effects (including vomiting, chills, and confusion) can make the childbirth experience perceived as traumatic [12,28].

In terms of childbirth type, the literature presents conflicting findings. While the majority of women who develop P-PTSD symptoms have had a spontaneous vaginal birth [29], other studies highlighted that an emergency caesarean section is associated with more intrusive P-PTSD symptoms compared to natural birth [30,31]. Preterm birth is also considered an important risk factor for P-PTSD; mothers of preterm infants exhibit higher rates of post-traumatic symptoms compared to mothers of full-term infants, both immediately after birth and one month postpartum [32]. In general, however, complications during childbirth that threaten the life of the mother and the child (e.g., hemorrhage, rupture of the uterus, or fetal distress) and require the use of invasive medical interventions (e.g., forceps, vacuum extraction, or episiotomy) are the main risk factors for perceiving childbirth as traumatic [12,21,33].

Postpartum risk factors include physical injury to either the mother and/or the infant as a result of childbirth. Specifically, mothers who suffer permanent physical injury to the reproductive system after childbirth often report alterations in their self-image and difficulties in accepting the physical changes that occur in the postpartum period [34]. Additionally, mothers of infants with neonatal complications (e.g., brain hemorrhages, neurological damage, or severe prematurity), which require admission to neonatal intensive care, report difficulties in feeling comfortable in their new role as mothers and perceive their relationship with their child as unnatural [35,36]. Infants’ characteristics also appear to affect maternal wellbeing. For example, a newborn’s difficult temperament coupled with the mother’s psychological distress may make the postpartum experience more challenging, thereby exacerbating P-PTSD symptoms [26].

Finally, perceived low social support from a partner, family members, and healthcare personnel is a significant risk factors throughout the entire perinatal period [37]. Specifically, the use of behaviours associated with obstetric mistreatment by healthcare personnel (e.g., physical and verbal abuse, violations of privacy, not providing information about the baby’s health, and administering medical care without consent) during pregnancy, childbirth, and the postpartum period can adversely affect woman’s emotional experience of childbirth, raising the risk of developing P-PTSD symptoms [38,39].

Understanding these factors is essential for developing screening tests for the early identification of women at risk of developing P-PTSD symptoms in order to intervene in time and prevent the consolidation of the disorder and its negative consequences on maternal wellbeing and the mother–child adjustment.

### 1.3. The Present Study

Self-report questionnaires are a very cost-effective and practical way of screening for early symptoms of maternal mental health problems in the postnatal period [40]. For this reason, several self-report questionnaires have been developed in the literature with the aim of early identification of women at risk of developing severe PTSD symptoms (i.e., Primary Care PTSD Screener for the DSM-5 [41]; Post-traumatic Stress Disorder Checklist for the DSM-5 [42]; Parental Stressor Scale: Neonatal Intensive Care Unit, PSS: NICU [43]). However, these questionnaires allow for the assessment of general PTSD symptoms that are not specifically associated with the traumatic experience of childbirth. Currently, to the best of our knowledge, The City Birth Trauma Scale (BiTS [44]) and the Perinatal PTSD questionnaire (PPQ [45]) are the only screening tools in the literature for identifying P-PTSD symptoms associated with traumatic childbirth.

The BiTS is a tool consisting of 29 items that allows for the identification of P-PTSD symptoms specifically associated with traumatic birth experiences in the post-partum period [15,44,46]. Compared to the BiTS, which was validated for the recognition of P-PTSD symptoms in women within 12 months after childbirth, the PPQ-II is a questionnaire that covers a broader perinatal period, from the immediate postpartum to 18 months. Specifically, this tool assesses P-PTSD symptoms that may result from both traumatic childbirth experiences and the child’s hospitalization during the short and long postnatal period [45]. Another strength of the instrument compared to BiTS is the number of items. In fact, with only 14 items, the PPQ-II allows for fast screening of P-PTSD symptoms. The original version of the PPQ aimed at recognizing P-PTSD symptoms in women at risk, such as mothers of premature children [47]. In the revised version of the questionnaire (PPQ-II), dichotomous items were replaced with Likert scale responses to facilitate the clinical interpretation of symptom severity [48]. Despite maintaining the three-factor structure of the original version, the PPQ-II includes items reflecting the four symptom clusters of the DSM-5, i.e., the re-experiencing of the traumatic event, cognitive and behavioural avoidance, negative mood and cognitive changes, and hyperarousal [6]. Because of its rapid administration and sensitivity in detecting symptoms of P-PTSD, the PPQ-II is an excellent perinatal screening tool that can be used by both psychologists and health professionals (such as midwives, gynecologists, psychologists, and nurses) to prevent the onset and consolidation of distress symptoms. The PPQ-II has demonstrated strong psychometric properties across multiple cultural validations (i.e., Africa [49]; Portugal [50]; China [51]; Spain, [52]; France, [53]; and Korea [54]).

Recently, the Italian version of the BiTS [55] has been validated, while the version of the PPQ-II is still unavailable. In order to fill this gap, the first aim of this study is to validate the Italian version of the PPQ-II by analysing its factorial structure and psychometric properties. In relation to this, it is hypothesized that the Italian version of the PPQ-II has adequate psychometric properties to identify women with P-PTSD symptoms effectively.

Additionally, there are currently no studies that comprehensively explore the effects of individual and environmental factors, along with factors related to pregnancy, childbirth, and the postpartum period, on the development of P-PTSD symptoms in the Italian population. Consequently, the second aim of this study is to explore risk and protective factors associated with the development of P-PTSD in a wide representative population of woman from 6 months after birth to the second year of the child’s life. It is hypothesized that the factors across various periods—including pre-pregnancy (i.e., low socioeconomic status, low educational level, history of miscarriages, and low social support), pregnancy (i.e., substance use, medical complications, being single or in the midst of a separation from a partner, and low social support), childbirth (i.e., emergency caesarean, prolonged labour, medical complications during childbirth, and low social support), and postpartum (i.e., maternal and neonatal medical complications, inadequate quality of health information about the baby from healthcare providers, and low social support)—are associated with higher P-PTSD symptoms following childbirth.

## 2. Materials and Methods

### 2.1. Participants and Procedure

Participants were N = 702 mothers (M age = 35.37 years, SD = 4.57) 6 to 24 months postpartum, who completed an online survey assessing sociodemographic and perinatal factors, postpartum post-traumatic stress symptoms related to childbirth, depression, anxiety, stress, and personality traits. The exclusion criteria for this study were refusal to participate by not giving consent and being under 18 years of age. In our sample there are not subgroups of high-risk women as indicated by the percentages of previous abortion, cesarean delivery, and preterm birth that are in line with the national average [56]. Recruitment occurred mainly via social media: the Qualtrics XM platform (Qualtrics Italy, Milan, Italy) was used to create and share the survey through an online anonymous link. Participants gave the consent to participate by clicking on the consent box before answering the survey, which included a detailed study description and ethical considerations. Participation was voluntary and not remunerated. The sample’s characteristics are illustrated in Table 1.

### 2.2. Measures

#### 2.2.1. Sociodemographic Information

A self-report questionnaire was used to record the following information: maternal age, maternal education level, socioeconomic status, marital status during pregnancy, child gender, and child age and birth order (i.e., primipara). Further, information on previous pregnancy-related experience was collected (i.e., previous abortions).

#### 2.2.2. Pregnancy-Related Factors

Women were asked to indicate the presence or absence of the following eight pregnancy risk factors: smoking, alcohol use, narcotics use, psychotropic drugs use, accidents, risk of abortion, and conjugal/financial problems. The total number of indicated factors was summed to create the Pregnancy Total Risk Factors (P-Risk) score that ranged from 0 to 8. Higher scores indicate the co-occurrence of greater risk factors.

The family and partner support during pregnancy was assessed with two specific questions (i.e., “How would you evaluate the support received from your partner/your family during pregnancy?”) with a 5-point Likert scale ranging from 0 = Absolutely inadequate to 4 = Absolutely adequate. Higher scores indicate a greater perception by the woman of having received high social support during the pregnancy.

#### 2.2.3. Childbirth-Related Factors

Mothers indicated the type of delivery (i.e., natural birth, instrumental birth, elective cesarean section, and emergency cesarean section), the duration of labour (i.e., more or less than 12 h labour), the presence of medical interventions (i.e., episiotomy and anesthesia), and whether there were any complications during the birth, including those related to the baby’s health. Moreover, they were asked to report whether they had the opportunity to have a trusted person present during delivery.

#### 2.2.4. Post-Partum Factors

The perceived family and partner support in the weeks immediately after childbirth was assessed with two specific questions (i.e., “How would you evaluate the support received from your partner/your family during the weeks immediately after childbirth?”) on a 5-point Likert scale ranging from 0 = Absolutely inadequate to 4 = Absolutely adequate. Higher scores indicate a greater perception by the woman of having received high social support during the post-partum period.

Mothers reported whether they did or did not receive information on the child’s health from health professionals after birth and if they experienced rooming-in (i.e., a practice adopted in hospitals where the newborn stays in the same room as the mother, rather than being placed in a separate nursery).

#### 2.2.5. Perinatal PTSD Symptoms

The Modified Perinatal PTSD Questionnaire (PPQ-II [57]) was administered to measure the PTSD symptoms related to childbirth. Specifically, it includes 14 items that aim to capture intrusiveness or re-experiencing (e.g., “Did you have bad dreams of giving birth or of your baby’s hospital stay?”), avoidance behaviours (e.g., “Did you try to avoid thinking about childbirth or your baby’s hospital stay?”), and hyperarousal or numbing of responsiveness (e.g., “Were you more irritable or angry with others than usual?”). Mothers provided responses related to their past experiences following a 5-point Likert scale from 0 = Never to 4 = Often for more than a month, with higher scores indicating higher levels of symptoms. The original PPQ-II showed high internal consistency with a Cronbach’s Alpha of 0.90 [57]. To achieve one of the aims of the current study, the scale was translated from English to Italian using a back-translation procedure. The translated scale was then checked for grammar, language, and comprehension. After correction by the experts, the final version of the scale was further translated into English and compared with the original scale to establish semantic, idiomatic, and conceptual consistency.

#### 2.2.6. Depressive, Anxiety, and Stress Symptoms

Mothers’ depressive, anxiety, and stress symptoms were assessed with the Italian-validated short version of the Depression Anxiety Stress Scales (DASS-21 [58]. The scale is composed of 21 items that evaluate three subscales: depression (e.g., “I felt that I had nothing to look forward to”), anxiety (e.g., “I was worried about situations in which I might panic and make a fool of myself”), and stress (e.g., “I found it difficult to relax”). Participants were asked to respond by thinking about their experiences over the past week following a 4-point Likert scale ranging from 0 = Did not apply to me at all to 3 = Applied to me very much or most of the time. Higher scores indicate higher levels of symptoms. The Italian adaptation has demonstrated good psychometric properties. In the current study, the Cronbach’s alpha reliability for the depression scale is 0.85, for the anxiety scale it is 0.78, and for the stress scale, it is 0.87. Following the previous validations [53], the scale was included in this study to determine the divergent validity of the PPQ-II.

#### 2.2.7. General Post-Traumatic Stress Symptoms

To investigate the convergent validity of the PPQ-II we used the Italian form of the Impact of Event Scale—Revised (IES-R) [59,60], a widely used measure of distress caused by traumatic events. We asked women to specifically reflect on their most recent childbirth experience. The IES-R consists of a total scale and three subscales: intrusion (e.g., “Any reminder brought back feelings about it); avoidance (e.g., “I avoided letting myself get upset when I thought about it or was reminded of it”); and hyperarousal (e.g., “I had trouble falling asleep”). Items are rated on a 5-point Likert scale from 0 = Not at all to 4 = Extremely. Higher scores indicate a greater risk of exhibiting PTSD symptoms. For the present study, we considered only the IES-R Total scale (α = 0.93).

#### 2.2.8. Personality Traits

To investigate the divergent validity of the PPQ-II, in line with the previous validations [53], we used the Italian version of the Big Five Inventory–10 (BFI–10) [61] to assess personality traits according to the five-factor approach [62]. It is composed of 10 items aimed at measuring 5 dimensions of personality. For the current study, we considered only the Openness to experience scale (e.g., “I see myself as someone who has an active imagination”) that reflects the degree of intellectual curiosity, creativity, and a preference for novelty and variety. Each item is rated on a 5-point Likert scale ranging from 1 = Strongly disagree to 5 = Strongly agree. Higher scores indicate a greater tendency to experience. The Sperman–Brown coefficient in the current study was 0.47, similar to the 0.50 of the validation study [61].

### 2.3. Analytic Plan

Descriptive statistics for the variables of interest were computed (see Table 1). For the validation of the Italian version of the PPQ-II we conducted an exploratory factor analysis (EFA), a confirmatory factor analysis (CFA), and explored divergent and concurrent validity.

To assess the suitability for conducting an EFA, we first examined the Kaiser–Meyer–Olkin’s [63] coefficient and Bartlett’s test of sphericity [64]. To determine the number of factors to retain, we applied the parallel analysis [65], in conjunction with the Guttman–Kaiser criterion (i.e., eigenvalue > 1.00). We then performed an EFA with oblique oblimin rotation as the factors were expected to be correlated. Factor loadings ≥ 0.30 and/or items with communalities ≥ 0.30 were considered meaningful [66], and items that did not meet these criteria or with balanced factor loadings across factors were excluded. The CFA, using maximum-likelihood estimation, was performed to test the stability of the model derived from the EFA. The model goodness of fit was evaluated using various indices that are widely accepted in the literature. These were selected for their robustness and reduced sensitivity to large sample sizes: the Comparative Fit Index (CFI), the Tucker–Lewis index (TLI), the Root-Mean-Square Error of Approximation (RMSEA), and the Standardized Root-Mean-Square Residual (SRMR). The fit indices were deemed acceptable based on the more stringent criteria (CFI and TLI ≥ 0.95, SRMR and RMSEA ≤ 0.06) proposed by Hu and Bentler [67], as well as considering the standard cut-off values (CFI and TLI ≥ 0.90, and SRMR and RMSEA ≤ 0.08) proposed by Kline [68].

Cronbach’s Alpha was used to estimate internal consistency of the Italian version of the PPQ-II. Convergent validity was assessed through bivariate associations between the PPQ-II and the IES-R, while divergent validity was assessed through bivariate associations with each scale of the DASS-21 and the Big Five Openness scale, using the Fisher’s Z Test to compare the correlations.

To examine the relationships between PPQ-II scores and various risk factors, with the goal of identifying aspects linked to an increased risk of developing P-PTSD, we performed bivariate analyses, independent *t*-tests, and one-way ANOVAs, with post hoc Tukey’s tests for significant results.

Finally, we determined the PPQ-II cutoff for high risk of P-PTSD calculating the 90th percentile and compared risk factors in mothers above and below this value using *t*-tests for continuous variables and Pearson’s chi-squared tests for categorical variables.

All statistical analyses were run using the statistical software R version 4.4.1 (see Supplementary Materials) [69,70,71,72,73].

## 3. Results

### 3.1. Exploratory Factor Analysis

Bartlett’s test of sphericity (χ^2^ = 597.88, df = 13, *p* < 0.001) and the Kaiser–Meyer–Olkin measure of sampling adequacy (KMO = 0.89) indicated that the data were suitable for EFA. Parallel analysis, along with the Guttman–Kaiser criterion, suggested 2 factors with eigenvalues above 1, accounting for 41% of the total variance. Four items with factor loadings < 0.30, with communalities < 0.30, and/or with balanced factor loadings across the two factors were dropped and excluded from further analysis (i.e., items 3, 6, 13, and 14 from the original version; see Supplementary Material S1), resulting in a 10-item scale. Then, the same extraction and rotation methods were applied to the remaining items, confirming the bi-factor structure, which explained 47% of the total variance. Factor 1, labeled “Arousal and Mood Alteration” (6 items), accounted for 26% of the variance, while Factor 2, labeled “Avoidance and Intrusion” (4 items), accounted for 21%. Item loadings, items included in each factor, communalities, and eigenvalues are reported in Table 2.

### 3.2. Confirmatory Factor Analysis

**Figure 1:**
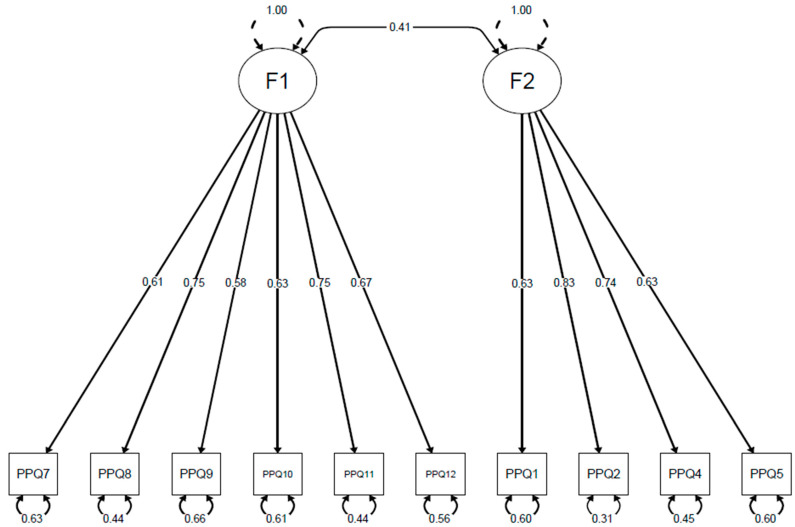
Confirmatory factor analysis of the Italian version of the PPQ-II. Note. F1: “Arousal and Mood Alteration”, F2: “Avoidance and Intrusion”. Depicted values show standardized factor loadings and residuals derived from the confirmatory factor analysis.

Next, we performed a CFA to evaluate the model’s goodness of fit of the PPQ-II bi-factor structure identified in the EFA. The results supported this solution, by demonstrating that the two factors, comprising the 10 items, yielded a good fit (CFI = 0.95; TLI = 0.94; RMSEA = 0.07; and SRMR = 0.04). All factor loadings were ≥0.30 (see Figure 1).

### 3.3. Internal Consistency

To evaluate the internal consistency of the Italian version of the PPQ-II, Cronbach’s Alpha was calculated for both the overall scale and its subscales. Alpha was 0.82 for the PPQ-II total scale, 0.82 for the PPQ-II Arousal and Mood Alteration, and 0.80 for the PPQ-II Avoidance and Intrusion, indicating a good internal consistency.

### 3.4. Convergent and Divergent Validity

The PPQ-II total score and each subscale’s score were positively associated with IES-R, suggesting good convergent validity (see Table 3).

The correlations between PPQ-II total score and DASS-21 subscales (i.e., depression, anxiety, and stress) were positive and significant (see Table 4). However, these correlations were significantly lower than the correlations observed between PPQ-II total score and IES-R indicating strong divergent validity, z = 8.43, *p* < 0.001; z = 10.83, *p* < 0.001; and z = 8.01, *p* < 0.001, for depression, anxiety, and stress, respectively. The correlations between PPQ-II subscales and DASS-21 subscales were significantly lower than correlations observed with IES-R as well, with values of z = 1.92, *p* < 0.05; z = 5.43, *p* < 0.001; and z = 1.92, *p* < 0.05 for PPQ-II Arousal and Mood Alteration, and z = 11.48, *p* < 0.001; z = 11.48, *p* < 0.001; and z = 10.77, *p* < 0.001 for PPQ-II Avoidance and Intrusion with depression, anxiety, and stress, respectively. Moreover, the PPQ-II total score and PPQ-II Arousal and Mood Alteration score were negatively associated with the Big Five Openness score. These correlations were significantly lower than the correlations between PPQ-II and IES-R, further supporting good divergent validity, with z = 18.06, *p* < 0.001 and z = 12.85, *p* < 0.001, for PPQ-II total score and PPQ-II Arousal and Mood Alteration, respectively.

### 3.5. P-PTSD Risk and Protective Factors

#### 3.5.1. Maternal Factors

Maternal age showed a slightly negative association with the PPQ-II total score, with younger mothers reporting higher scores (see Table 5).

Primipara mothers reported higher PPQ-II total scores (M = 14.49, SD = 8.06) than those with more children (M = 11.62, SD = 7.38), t (700) = 3.98, *p* < 0.001. The PPQ-II scores were similar across maternal education and SES and between mothers who had experienced previous abortions and those who had not, F(1, 700) = 2.80, *p* = 0.094, F(3, 666) = 1.98, *p* = 0.12, t(700) = 1.03, *p* = 0.305, respectively.

#### 3.5.2. Pregnancy-Related Factors

Mothers who encountered more pregnancy risk factors and that received less support from their partners and family during pregnancy reported higher PPQ-II scores (see Table 5).

#### 3.5.3. Birth-Related Factors

Mothers who experienced labor lasting longer than 12 h reported higher PPQ-II total scores (M = 15.40, SD = 8.73) than mothers who had labor lasting less than 12 h (M = 12.80, SD = 7.45), t(631) = 3.93, *p* < 0.001. Comparing different types of delivery (i.e., natural birth, instrumental delivery with forceps or vacuum, elective cesarean section, and emergency cesarean section), there was a significant difference in PPQ-II total scores, F(3, 698) = 8.06, *p* < 0.001. Post hoc Tukey’s comparisons revealed that mothers who experienced an emergency cesarean section reported higher PPQ-II scores (M = 16.87, SD = 9.76) than those who had a natural birth (M = 12.95, SD = 7.47), *p* < 0.001.

A significant difference was also found in PPQ-II total scores between mothers, excluding those who had cesarean section, who experienced anesthesia during labor (M = 14.01, SD = 7.52) and those who did not (M = 12.56, SD = 7.55), t(509) = 2.11, *p* = 0.035. Mothers who had complications during delivery reported higher PPQ-II total scores (M = 17.29, SD = 9.04) than those who did not (M = 13.51, SD = 7.83), t(688) = 3.67, *p* < 0.001. Moreover, mothers who did not have a trusted person with them during delivery had significantly higher PPQ-II total scores (M = 16.05, SD = 8.48) than those with a trusted person during delivery (M = 13.15, SD = 7.71), t(700) = 4.17, *p* < 0.001. Levels of PPQ-II were similar among mothers who experienced episiotomy and mothers who did not, t(684) = 1.69, *p* = 0.092.

Regarding infant-related factors, levels of PPQ-II were similar for mothers of preterm and full-term children, t(700) = 0.85, *p* = 0.393, and for mothers who reported or not infant complications after birth, t(647) = 0.98, *p* = 0.328.

#### 3.5.4. Post-Partum Factors

Mothers who reported a lack of information about their infant’s health from medical staff showed higher PPQ-II scores (M = 19.04, SD = 8.72) than mothers who were informed (M = 12.80, SD = 7.42), t(699) = 8.12, *p* < 0.001. Similarly, mothers who did not room-in with their infants after birth showed higher PPQ-II scores (M = 16.07, SD = 8.90) than those who did (M = 12.96, SD = 7.43), t (700) = 4.75, *p* < 0.001. Finally, mothers who reported more support from their partners and family during the post-partum period reported lower PPQ-II scores (see Table 5).

#### 3.5.5. Identifying and Characterising Mothers at High-Risk of P-PTSD

To identify mothers at higher risk of P-PTSD, we set a cut-off score at the 90th percentile, which corresponded to a score of 25. Using this threshold, 71 mothers (i.e., 10.11% of the total sample) were classified as high-risk. Analysis showed that high-risk mothers faced more risk factors during pregnancy and received less family support during this period. There was also a trend suggesting that high-risk mothers were younger and less supported by their partner during pregnancy. Furthermore, they were more likely to experience labor lasting over 12 h, require emergency cesarean sections, use anesthesia and encounter complications during delivery. High-risk mothers were also more likely to lack the presence of a trusted person during delivery, primipara, and receive inadequate information about their infant’s health from medical staff. They were less likely to room-in with their newborns after birth and received less support from both their partners and families during the post-partum period. Detailed results from *t*-test and chi-squared analyses are provided in the Supplementary Materials (see Tables S2 and S3).

## 4. Discussion

The aim of this study was to validate the Italian version of the PPQ-II, a tool designed to quickly identify early symptoms of P-PTSD in women during the postnatal period. An additional key objective was to identify maternal risk and protective factors, as well as those related to the perinatal period, that may contribute to the development of P-PTSD symptoms. The integration of screening tools like the PPQ-II into the daily clinical practice of obstetrics and gynecology departments is a crucial strategy for early intervention. By combining such tools with the identification of perinatal risk and protective factors through clinical observation, it is possible to prevent traumatic experiences during childbirth and improve maternal psychological well-being.

### 4.1. The Italian Validation of the PPQ-II

The primary objective of this study was to validate the Italian version of the PPQ-II. To assess the PPQ-II as a screening tool for general P-PTSD symptoms, we chose to conduct the analysis on a non-targeted sample. Consistent with previous validation studies, the results demonstrated the strong reliability of the scale, confirming that the items in the PPQ-II effectively assess symptoms associated with PTSD related to traumatic childbirth [51,52,74]. Moreover, our results indicate that a Likert scale is an effective method for assessing general P-PTSD symptoms, as it provides a detailed and accurate measurement [50]. This result supports the conclusion drawn by Callahan et al. [23] and other validation studies [51,74], who found that employing a Likert scale improves the internal consistency of measurement tools.

While the original version [23] included 14 items grouped in 3 factors corresponding to the DSM-IV criteria (intrusion, avoidance, and hyperarousal), the Italian version of the PPQ-II consists of 10 items clustered into 2 factors: “Arousal and Mood Alterations” and “Avoidance and Intrusion”. The main difference compared to the original version is the integration of the “Intrusion” factor into the “Avoidance” factor. This integration may reflect the fact that women who experience intrusive memories of childbirth often adopt avoidance behaviours to cope with trauma-related stimuli [50]. Notably, Item 1, “Did you have bad dreams of giving birth or of your baby’s hospital stay?”, is the only intrusive symptom carried over from the original scale and included under the “Avoidance and Intrusion” factor (see Appendix A and Appendix B). Its inclusion in the questionnaire may be explained by the fact that nightmares are difficult to avoid, making it the only item capable of detecting intrusive symptoms of P-PTSD. In line with previous validations, it is noteworthy that the “Arousal and Mood Alterations” factor encompasses symptoms such as emotional numbing and hypervigilance [74]. This is consistent with the literature, which explains that trauma activates the primitive nervous system, such as the limbic system and the amygdala, involved in the perception of danger and the “fight or flight” response, aimed at guaranteeing the individual’s survival [75]. This constant activation results in increased energy consumption, which can deplete cognitive resources and impair the functioning of the prefrontal cortex, responsible for executive functions, emotional regulation, planning, and concentration. These processes explain the co-occurrence of mood and physiological disturbances typical of PTSD, such as difficulty concentrating, irritability, and a sense of emotional disconnection from others [76].

Although the tool’s two-factor structure is consistent with the French [53], Portuguese [50], and Turkish validations [74], it has a theoretical weakness, as it does not fully reflect the grouping of PTSD diagnostic criteria in the DSM-IV, upon which the original version of PPQ was based. However, our findings confirm that the factor structure of the scale makes the PPQ-II valid for detecting the overall symptoms of P-PTSD according to the updated DSM-5 criteria. Given that the PPQ-II is intended as a screening tool for the global symptoms of P-PTSD, it is recommended to use the total score in clinical practice, rather than relying on the individual subscales [74].

As with the Portuguese version, items 3, 6, and 14 (“Did you have any sudden feelings as though your baby’s birth was happening again?”, “Were you unable to remember parts of your baby’s hospital stay?”, and “Did you feel more guilt about the childbirth than you felt you should have felt?”) were removed due to their low sensitivity in detecting P-PTSD symptoms [50]. These results could be interpreted because both the Italian and Portugues versions of the tool were validated on general populations and not on high-risk samples as the original version (i.e., mothers of premature infants), who may develop a different symptomatology. Additionally, in our validation, item 13 (“Did you feel more jumpy?”) was excluded due to the question’s interpretative ambiguity, which could be attributed to a possible different cultural misinterpretation of the Italian translation of the word “jumpy”.

The higher correlation between the PPQ-II and the IES suggests that they are measuring similar hypothetical constructs, confirming the concurrent validity of the tool. The correlations among the PPQ-II and the DASS-21 scales (i.e., anxiety, depression, and stress) are significantly lower compared to those with the IES, suggesting that these tools measure symptoms associated with different psychological constructs. These results further confirm that women in the postpartum period may exhibit P-PTSD symptoms alongside anxiety and mood disorders [33]. The validity of the tool is further supported by the absence of a correlation between the PPQ-II and the Big Five Openness scale. The results of the divergent validity analyses indicate that the two instruments assess separate psychological constructs, confirming that the PPQ-II is a reliable tool to evaluate symptoms of psychological distress related to traumatic childbirth, which are independent from the personality trait of openness to new experiences.

### 4.2. Perinatal Risk and Protective Factors

The second aim of our study was to identify the risk and protective factors associated with the onset of P-PTSD symptoms throughout the entire perinatal period. This represents one of the key strengths of the study, as there is a lack of studies that simultaneously examine the role of maternal, pregnancy, childbirth, and postpartum factors on maternal P-PTSD.

#### 4.2.1. Maternal Factors

Younger mothers are more likely to develop P-PTSD symptoms, which helps identify a particularly vulnerable group. While it is known that women aged 23 to 35 are at higher risk for exhibiting general PTSD symptoms, research on the relationship between maternal age and specific symptomatology related to the traumatic childbirth experience remains limited [24,77]. It is known that younger mothers might experience a lack of emotional resources, limited support networks, and stressful life experiences, making them more vulnerable to experiencing childbirth as a traumatic event [78,79].

While general PTSD symptoms have been reported to decrease over time, our findings suggest that P-PTSD symptoms did not vary according to the child’s age [6]. Similarly, in contrast with previous findings, we did not find an effect of maternal SES and history of previous abortions. The lack of effect of maternal SES could be explained by the fact that the Italian healthcare system provides free access to routine gynecological visits during pregnancy for the entire population, reducing treatment disparities based on socio-economic status [12,13,23]. Moreover, the small number of women in our sample who reported having a history of abortions might be the reason why we did not find evidence of the role of history of previous abortions in increasing the risk of perceiving childbirth as traumatic, as is reported by previous studies [49,80].

#### 4.2.2. Pregnancy-Related Factors

Consistent with previous American and European studies that reported that women who smoked, consumed alcohol or drugs, or have experienced adverse events such as marital conflicts, financial difficulties, and relocations during pregnancy were more likely to have difficulty coping with environmental challenges, our findings showed a higher risk for these women of experiencing childbirth as traumatic [5,12]. Furthermore, women who benefit from adequate social support from their partner and family members during pregnancy have a lower probability of developing severe symptoms of P-PTSD, highlighting how an effective social support network is an important protective factor in promoting maternal well-being during pregnancy [1,81].

#### 4.2.3. Birth-Related Factors

Consistent with European and American literature, our findings confirmed that women who experience prolonged labor, lasting over 12 h, are more likely to develop P-PTSD symptoms, often due to prolonged physical pain [21,24]. While some studies suggested anesthesia as a protective factor by reducing physical and psychological suffering [12,26], our results showed that anesthesia could be a risk factor for P-PTSD. As reported by some studies conducted in Europe, these findings may be due to anesthesia’s side effects (e.g., vomiting, intense cold, and loss of control), which can contribute to a traumatic childbirth experience [28,52]. Exploring anesthesia’s timing and side effects could provide more insight into its role in P-PTSD development. As labor progresses, delayed anesthesia may reduce its effectiveness and increase side effects, making the experience more stressful. Developing a plan for optimal anesthesia timing and informing the patient of potential side effects is crucial for improving the childbirth experience.

An additional risk factor for P- PTSD, as already highlighted in the American validation of the PPQ -II, is unplanned cesarean delivery [53]. While necessary for medical reasons, it increases the perception of childbirth as life-threatening for both mother and child due to its unexpected and invasive nature [11,28]. We also found a higher prevalence of P-PTSD in first-time mothers. This is clinically significant, as previous studies have shown that fear, anxiety, and dissociation during the first childbirth not only affect the mother’s psychological well-being but also increase the risk of avoidance behaviors related to the trauma, such as medical visits and future pregnancies [36,51].

Among protective factors, having a trusted person, such as a partner or family member, present during labor can reduce the risk of perceiving the experience as traumatic [2], emphasizing the importance of social support not only during pregnancy, but also during childbirth.

Contrary to the literature, episiotomy was not a risk factor for P-PTSD. A British study suggested that episiotomy may increase feelings of helplessness during childbirth, potentially making the experience more negative [82]. Exploring women’s perceptions of episiotomy could help explain this discrepancy.

Unexpectedly, preterm birth and neonatal infant complications were not specific risk factors for P-PTSD in our sample. While previous validations of the PPQ-II based on high-risk samples found such associations (i.e., Portugal, America, Spain, Korea, and Turkey), our study found no significant links in a general population with few premature infants and postnatal complications [50,53,66].

#### 4.2.4. Post-Partum Factors

According to Spanish validation, our findings confirmed that the quality of medical care, often focused on ensuring biological survival at the expense of maternal psychological well-being, influences a woman’s perception of the childbirth experience [83]. Specifically, a lack of information from healthcare personnel about the child’s health status increases the risk of P-PTSD, while the possibility of having the baby in the same room as the mother (i.e., rooming-in) and the quality of social support are represented as protective factors. Indeed, women who reported inadequate communication with healthcare personnel may feel abandoned, insecure, and fearful due to the uncertainty of not knowing their baby’s health condition [3]. In contrast, the practice of rooming-in, particularly if intentionally planned by the mother before childbirth, may reduce maternal stress and promote a secure mother–child affective bond [15]. Finally, having supportive individuals, such as partners and family members, can provide essential practical and emotional support, which helps reduce the risk of developing P-PTSD and promotes a positive adaptation to the postpartum period [13,79,84].

### 4.3. Limits and Suggestions for Future Studies

This study has some limitations that should be considered for future research. Firstly, as a cross-sectional study, it could not examine the development of P-PTSD and its causal relationship between the experience of traumatic childbirth, maternal well-being, and the quality of the mother–child affective bond. Future longitudinal studies using a mixed-methods approach, including questionnaires and observational data, would help explore the evolution of P-PTSD and its short- and long-term effects on maternal well-being and the mother–child relationship. Following this perspective, it is recommended to analyze the phenomenon at different time periods, such as during pregnancy and at 6, 12, and 24 months postpartum. Another limitation is the lack of data on the quality of the childbirth experience as perceived by the woman and how this might predict P-PTSD development. Future research should focus on developing tools to assess maternal perception of childbirth to better understand its impact on maternal mental health. Additionally, it is known that PTSD symptoms can develop in individuals who witness traumatic events. This often-overlooked aspect deserves attention, as the traumatic nature of the childbirth experience can also impact the mental health of others involved, such as fathers and healthcare professionals. Finally, the absence of high-risk populations from the sample limits the generalizability of the results. Therefore, it is necessary to validate the Italian version of PPQ-II for these populations (e.g., women who have experienced domestic abuse and trauma and mothers of premature infants) to explore potential symptomatic differences and improve screening tools.

### 4.4. Implications for Clinical Practice and Research

This study has important clinical implications. Consistent with previous findings across different countries, the PPQ-II is a brief, fast, and simple screening tool that healthcare professionals can incorporate into the routine practices of gynecology and obstetrics departments without specific training. The time available to administer the PPQ-II may be limited by the high number of patients in maternity care units. The integration of an online version of the tool into screening protocols may be an excellent solution for the identification and monitoring of P-PTSD symptoms throughout the perinatal period. A cut-off score of 25 has been established to facilitate clinical interpretation, with scores above this threshold indicating an elevated risk of P-PTSD.

Additionally, this study contributed to identifying maternal risk factors and those related to the entire perinatal period, which are essential for identifying women, even during pregnancy, who are more at risk for perceiving childbirth as a traumatic experience. Healthcare professionals play a critical role in recognizing and preventing perinatal mental health issues, as they support women throughout pregnancy and the puerperium. However, lack of training on risk factors for perinatal mental disorders may limit identifying women at risk of developing P-PTSD. Therefore, it would be beneficial to develop national guidelines that include continuing education for healthcare providers, including gynecology and obstetrics students, focusing on the administration and scoring of the PPQ-II, as well as raising awareness of risk and protective factors related to traumatic childbirth. It is important to emphasize that the PPQ-II is a screening tool designed to identify women who are more vulnerable to developing P-PTSD. Following this, future studies should focus on the development of interview and clinical observation methods and the effectiveness of psychological therapies that are useful in the diagnostic assessment and reduction of P-PTSD symptoms (e.g., Cognitive Behavioural Therapy, eye movement desensitization, and exposure therapy). As traumatic experiences negatively affect the individual’s psychophysiological system, future research should investigate how neurofeedback may reduce physiological symptoms of P-PTSD in a population that cannot benefit from an integrated clinical approach of psychotherapy and medication. Finally, because of the economic impact of untreated P-PTSD symptoms on the health care system, national policy guidelines are necessary to establish screening and social support programs for the promotion of the psychological well-being of the entire family system during pregnancy and throughout the perinatal period.

## Figures and Tables

**Table 1:** Sociodemographic and clinical characteristics of the sample (N = 702).

Variables	M (SD; Range)/n (%)
Sociodemographic information	
Maternal age	35.37 (4.58; 20.00–58.00)
Maternal education	
Elementary/Middle School	40 (6%)
High School	232 (33%)
University	330 (48%)
Postgraduate Degree	100 (13%)
Socioeconomic status	
<EUR 750	10 (1%)
EUR 750 < x < EUR 2150	244 (35%)
EUR 2150 < x < EUR 4950	395 (56%)
>EUR 4950	21 (3%)
Previous Abortions	135 (19%)
Primipara	548 (78%)
Marital status during pregnancy	
Married/cohabitating/in a relationship	695 (99%)
Child gender (M)	350 (50%)
Child age	13.68 (5.04; 6.00–24.00)
Pregnancy-related factors	
Family support during pregnancy	4.08 (0.86;5.00–1.00)
Partner support during pregnancy	4.32 (0.83; 5.00–1.00)
Pregnancy total risk factors (P-Risk)	0.33 (0.58; 3.00–0.00)
Childbirth-related factors	
Duration of labor (>12 h)	216 (31%)
Type of Delivery	
Natural Birth	449 (65%)
Instrumental Birth	53 (8%)
Elective Cesarean Section	69 (10%)
Emergency Cesarean Section	121 (17%)
Anesthesia	193 (38%)
Episiotomy	118 (17%)
Complications during delivery	66 (10%)
Trusted person presence during delivery	530 (75%)
Full-term delivery	644 (92%)
Infant’s health complications	140 (20%)
Post-partum related factors	
Post-partum family support	3.99 (0.99; 5.00–1.00)
Post-partum partner support	4.22 (0.96; 5.00–1.00)
Child’s health information	581 (83)
Rooming-in	498 (71)

**Table 2:** Exploratory factor analysis: factor loadings and communalities for each item (N = 702).

	F1	F2	h^2^
PPQ 1		0.64	0.41
PPQ 2		0.82	0.66
PPQ 4		0.76	0.58
PPQ 5		0.62	0.39
PPQ 7	0.57		0.35
PPQ 8	0.72		0.55
PPQ 9	0.53		0.31
PPQ 10	0.68		0.42
PPQ 11	0.79		0.60
PPQ 12	0.64		0.42
Eigenvalues	4.88	1.98	
Cumulative variance	0.26	0.47	

Note. The first column presents the 10 items of the Italian version of the PPQ-II (items 3, 6, 13, and 14 of the original version were eliminated). Factor 1 = arousal and mood alteration; Factor 2 = avoidance and intrusion. h^2^ = communality.

**Table 3:** Convergent validity: correlations between PPQ-II (total and subscales) and IES total score.

	Mean (SD)	1	2	3	4
1. PPQ-II Tot.	23.86 (8)	-			
2. PPQ-II Ar.	17.33 (5.97)	0.90 ***	-		
3. PPQ-II Av. and Intr.	6.54 (3.65)	0.71 ***	0.33 ***	-	
4. IES Tot.	1.79 (0.68)	0.74 ***	0.57 ***	0.68 ***	-

Note. PPQ-II tot. = Modified Perinatal Post-Traumatic Stress Disorder Questionnaire total score; PPQ-II Ar. = PPQ-II Arousal; PPQ-II Av. and Intr. = PPQ-II Avoidance and Intrusion; IES Tot. = IES total score. *** *p* < 0.001.

**Table 4:** Divergent validity: correlations between PPQ-II, DASS-21, and Big Five-10 (Openness).

	Mean (SD)	1	2	3	4	5	6	7
1. PPQ-II Tot.	23.86 (8)	-						
2. PPQ-II Ar.	17.33 (5.97)	0.90 ***	-					
3. PPQ-II Av. and Intr.	6.54 (3.65)	0.71 ***	0.33 ***	-				
4. Big Five Openness	4.14 (1.04)	−0.10 *	−0.10 *	−0.05	-			
5. DASS-21 Depression	8.13 (7.74)	0.43 ***	0.49 ***	0.16 ***	−0.09 *	-		
6. DASS-21 Anxiety	5.83 (6.51)	0.31 ***	0.32 ***	0.16 ***	−0.08	0.63 ***	-	
7. DASS-21 Stress	12.63 (8.89)	0.45 ***	0.49 ***	0.20 ***	−0.09 *	0.75 ***	0.64 ***	-

Note. PPQ-II tot. = Modified Perinatal Post-Traumatic Stress Disorder Questionnaire total score; PPQ-II Ar. = PPQ-II Arousal; PPQ-II Av. and Intr. = PPQ-II Avoidance and Intrusion. * *p* < 0.05; *** *p* < 0.001.

**Table 5:** Bivariate associations among PPQ-II (total score), sociodemographic information, pregnancy-related risk factors, and partner and family support during pregnancy and in the post-partum period. (N = 573).

	1	2	3	6	7	8	9	10
1. PPQ-II tot	-							
2. Child Age	0.04	-						
3. Maternal Age	−0.11 **	0.11 **	-					
6. Pregnancy risk factors	0.10 *	−0.02	−0.04	-				
7. P. support pregnancy	−0.16 ***	0.02	0.02	−0.18 ***	-			
8. F. support pregnancy	−0.19 ***	0	−0.05	−0.21 ***	0.39 ***	-		
9. Post-partum P. support	−0.22 ***	0.01	0.02	−0.13 **	0.63 ***	0.25 ***	-	
10. Post-partum F. support	−0.27 ***	0.01	−0.02	−0.20 ***	0.26 ***	0.64 ***	0.35 ***	-

Note. PPQ-II tot. = Modified Perinatal Post-Traumatic Stress Disorder Questionnaire total score; SES = socioeconomic status; P. support pregnancy = partner support during pregnancy; F. support pregnancy = family support during pregnancy; Post-partum P. support = post-partum partner support; Post-partum F. support = post-partum family support. * *p* < 0.05; ** *p* < 0.01; *** *p* < 0.001.

## Data Availability

The original contributions presented in this study are included in the article and Supplementary Material. Further inquiries can be directed to the corresponding authors.

## Supplementary Materials

The following supporting information can be downloaded at: https://www.mdpi.com/article/10.3390/jcm14030704/s1, Table S1: Exploratory factor analysis: factor loadings and communalities for each item; Table S2: Independent *t*-test of risk factors between low- and high-risk mothers for P-PTSD; Table S3: Chi-squared tests of risk factors between low- and high-risk mothers for P-PTSD; Table S4: The comparison between Italian validation of PPQ-II and previous ones; Figure S1: Flowchart of the Italian PPQ-II validation process. References [69,70,71,72,73] are cited in the Supplementary materials.

## Appendix A

**Table A1:** The Italian translation of the original version of the PPQ-II.

Modified Perinatal Post-Traumatic Stress Disorder Questionnaire (PPQ-II)	Italian Version of Modified Perinatal Post-Traumatic Stress Disorder Questionnaire (PPQ-II)
Did you have bad dreams of giving birth or of your baby’s hospital stay?	Le è capitato di fare incubi ricorrenti sul parto o sul periodo di ricovero del suo bambino in ospedale?
2. Did you have upsetting memories of giving birth or of your baby’s hospital stay?	2. Le è capitato di avere ricordi sconvolgenti legati al parto o al ricovero del suo bambino in ospedale?
3. Did you have any sudden feelings as though your baby’s birth was happening again?	3. Le è capitato di rivivere o sentire improvvisamente come se la nascita del suo bambino stesse accadendo di nuovo?
4. Did you try to avoid thinking about childbirth or your baby’s hospital stay?	4. Ha cercato di evitare di pensare al parto o al ricovero in ospedale del bambino?
5. Did you avoid doing things that might bring up feelings you had about childbirth or your baby’s hospital stay (e.g., not watching a TV show about babies)?	5. Le è capitato di evitare di fare cose che potrebbero rievocarle le emozioni che ha provato durante il parto o il ricovero del suo bambino in ospedale (per esempio non guardare un programma televisivo sui bambini)?
6. Were you unable to remember parts of your baby’s hospital stay?	6. Le è capitato di non essere in grado di ricordare qualcosa circa il ricovero del suo bambino in ospedale?
7. Did you lose interest in doing things you usually do (e.g., did you lose interest in your work or family)?	7. Le è capitato di nutrire scarso interesse per le attività e i passatempi che era solita fare prima del parto (per esempio ha perso interesse nel suo lavoro o nella sua famiglia)?
8. Did you feel alone and removed from other people (e.g., did you feel like no one understood you)?	8. Si è sentita sola e distante dalle altre persone? (per esempio le è sembrato di non essere capita da nessuno)
9. Did it become more difficult for you to feel tenderness or love with others?	9. Le è diventato più difficile sentire amore e tenerezza verso gli altri?
10. Did you have unusual difficulty falling asleep or staying asleep?	10. Ha avuto difficoltà ad addormentarsi o a riposare a sufficienza?
11. Were you more irritable or angry with others than usual?	11. Si è sentita più tesa, agitata e facilmente irritabile del solito con gli altri?
12. Did you have greater difficulties concentrating than before you gave birth?	12. Ha riscontrato maggiori difficoltà nel concentrarti rispetto a prima del parto?
13. Did you feel more jumpy (e.g., did you feel more sensitive to noise, or more easily startled)?	13. Si è sentita più eccitabile? (per esempio, più sensibile ai rumori, o si sorprendeva più facilmente)
14. Did you feel more guilt about the childbirth than you felt you should have felt?	14. Si è sentita più colpevole a proposito della nascita del bambino rispetto a come pensava si sarebbe dovuta sentire?

## Appendix B

**Table A2:** Italian version of the Modified Perinatal Post-Traumatic Stress Disorder Questionnaire (PPQ-II).

Scelga con attenzione la risposta che meglio descrive la sua situazione segnando il numero che corrisponde alla sua risposta								
0	1	2	3	4				
per niente	una o due volte	qualche volta	spesso, ma per meno di un mese	spesso, per più di un mese				
Le è capitato di fare incubi ricorrenti sul parto o sul periodo di ricovero del suo bambino in ospedale?				0	1	2	3	4
2. Le è capitato di avere ricordi sconvolgenti legati al parto o al ricovero del suo bambino in ospedale?				0	1	2	3	4
3. Ha cercato di evitare di pensare al parto o al ricovero in ospedale del bambino?				0	1	2	3	4
4. Le è capitato di evitare di fare cose che potrebbero rievocarle le emozioni che ha provato durante il parto o il ricovero del suo bambino in ospedale (per esempio non guardare un programma televisivo sui bambini)?				0	1	2	3	4
5. Le è capitato di nutrire scarso interesse per le attività e i passatempi che era solita fare prima del parto (per esempio ha perso interesse nel suo lavoro o nella sua famiglia)?				0	1	2	3	4
6. Si è sentita sola e distante dalle altre persone? (per esempio, le è sembrato di non essere capita da nessuno)				0	1	2	3	4
7. Le è diventato più difficile sentire amore e tenerezza verso gli altri?				0	1	2	3	4
8. Ha avuto difficoltà ad addormentarsi o a riposare a sufficienza?				0	1	2	3	4
9. Si è sentita più tesa, agitata e facilmente irritabile del solito con gli altri?				0	1	2	3	4
10. Ha riscontrato maggiori difficoltà nel concentrarti rispetto a prima del parto?				0	1	2	3	4

Note. The item numbers in the Italian validation do not match those in the original version. The scoring of the Italian version of the PPQ-II is based on a total score, which is the sum of the items from each factor. This score ranges from 0 to 50, with higher scores indicating more severe P-PTSD symptoms. A cut-off score greater than or equal to 25 indicates clinically significant distress.
