# Supplementary material for: The Validation of the Perinatal Post-Traumatic Questionnaire in the Italian Population: Risk and Protective Factors
Source: J Clin Med. 2025 Jan 22;14(3):704. doi: 10.3390/jcm14030704 (PMC11818509; doi:10.3390/jcm14030704)
Supplement: Supplementary file 1 [file jcm-14-00704-s001.zip › jcm-3400048-supplementary.pdf]

The Validation of the Perinatal Post-traumatic Questionnaire in the Italian Population: Risk and Protective Factors

Supplementary Material

11/28/2024

Contents

1. **Exploratory Factor Analysis** 2

1.1 Table S1. Factor Loadings and Communalities for Each Item…………………………2

2. **Identifying and Characterizing Mothers at High-Risk of P-PTSD** 3

2.1 Table S2. Independent T-Test of Risk Factors…………………………………………3

2.2 Table S3. Chi-squared Tests of Risk Factors……………………………………………...4

3. **The comparison between different versions of the PPQ-II** 6

3.1 Table S4. Comparison of the Italian PPQ-II Validation and Previous Versions............6

4. **The validation process of the Italian version of PPQ-II** 7

4.1 Flowchart of the Italian PPQ-II validation process……………………………………7

Used R packages……………………………………………………………………………..8

1. **Exploratory Factor Analysis**

Table S1.

Exploratory Factor Analysis: Factor Loadings and Communalities for Each Item (Including Removed Items)

	F1	F2	h^2
**PPQ 1**	**-0.01**	**0.63**	**0.40**
**PPQ 2**	**-0.01**	**0.80**	**0.64**
PPQ 3	0.01	0.41	0.18
**PPQ 4**	**-0.01**	**0.76**	**0.58**
**PPQ 5**	**-0.01**	**0.66**	**0.42**
PPQ 6	0.16	0.37	0.21
**PPQ 7**	**0.57**	**0.06**	**0.35**
**PPQ 8**	**0.72**	**0.05**	**0.55**
**PPQ 9**	**0.53**	**0.06**	**0.31**
**PPQ 10**	**0.68**	**-0.08**	**0.42**
**PPQ 11**	**0.79**	**-0.05**	**0.59**
**PPQ 12**	**0.66**	**0.01**	**0.44**
PPQ 13	0.48	0.05	0.25
PPQ 14	0.32	0.39	0.36
Cumulative variance	0.22	0.41	

In Table S1, the factor loadings, communalities and total variance of the bi-factor model are presented prior to the removal of ambiguous items. Items 3, 6 and 13 were excluded due to their communalities being below .30. Although item 14 met the communality criterion (i.e., > .30), it was removed because its factor loadings were similarly distributed across both factors.

2. Identifying and Characterizing Mothers at High-Risk of P-PTSD

Table S2.

Independent T-Test of Risk Factors Between Low- and High-risk Mothers for P-PTSD

	Low-risk Group (*n* = 631)	High-risk Group (*n* = 71)			
	M(SD)	*M(SD)*	*t*	*df*	*p*
Maternal Age	35.48(4.59)	34.38(4.41)	1.93	700	.054
Child Age	13.59(5.04)	14.51(5.01)	1.46	700	.145
Pregnancy Risk Factors	**.32(.57)**	**.49(.63)**	**2.07**	**685**	**.042**
Family Support (Pregnancy)	**4.12(.83)**	**3.81(1.03)**	**2.38**	**699**	**.019**
Partner Support (Pregnancy)	4.34(.81)	4.14(1)	1.90	697	.058
Family Support (Postpartum)	**4.06(.93)**	**3.44(1.31)**	**3.88**	**700**	**< .001**
Partner Support (Postpartum)	**4.31(.91)**	**3.80(1.23)**	**4.24**	**696**	**< .001**

Note. Low-risk mothers are those who scored below the 90th percentile cut-off score of 25, while high-risk mothers are those who scored at or above the 90th percentile cut-off score of 25.

Table S3.

Chi-squared Tests of Risk Factors Between Low- and High-risk Mothers for P-PTSD

	Low-risk Group		High-risk Group				
	n(%) Observed	n(%) Expected	n(%) Observed	n(%) Expected	χ2	*df*	*p*
**Sociodemographic Information**							
Maternal Education					8.01	4	.091
Elementary School	1(.16%)	.89(.14%)	0(0%)	.10(.14%)			
Middle School	38(6.02%)	35.06(5.56%)	1(1.41%)	3.94(5.56%)			
High School	214(33.91%)	208.54(33.05%)	18(25.35%)	23.46(33.05%)			
University	294(46.59%)	296.62(47.01%)	36(50.70%)	33.38(47.01%)			
Postgraduate Degree	84(13.31%)	89.89(14.25%)	16(22.54%)	10.11(14.25%)			
SES					2.37	3	.500
< 750€	10(1.67%)	8.96(1.49%)	0(0%)	1.04(1.49%)			
750€< x< 2150€	214(35.67%)	218.51(36.42%)	30(42.86%)	25.49(36.42%)			
2150€< x< 4950€	357(59.50%)	353.73(58.96%)	38(54.29)	41.27(58.96%)			
> 4950€	19(3.17%)	18.81(3.13%)	2(2.86%)	2.19(3.13%)			
Previous Abortions					.47	1	.494
No	507(80.35%)	376.82(65.88%)	60(84.51%)	40.19(65.88%)			
Yes	124(19.65%)	195.19(34.12%)	11(15.49%)	20.82(34.12%)			
Primipara					**5.97**	**1**	**.015**
No	147(23.30%)	138.43(21.94%)	7(9.86%)	15.58(21.94%)			
Yes	484(76.70%)	492.58(78.06%)	64(90.14%)	55.43(78.06%)			
**Birth-related Factors**							
Duration of Labor					**7.57**	**1**	**< .01**
< 12 hours	387(67.66%)	376.82(65.88%)	30(49.18%)	40.19(65.88%)			
> 12 hours	185(32.32%)	195.19(34.12%)	31(50.82%)	20.82(34.12%)			
Type of Delivery					**33.22**	**3**	**< .001**
Natural Birth	428(67.83%)	412.58(65.39%)	31(43.66%)	46.42(65.39%)			
Instrumental Birth	46(7.29%)	47.64(7.55%)	7(9.86%)	5.36(7.55%)			
Elective Cesarean Section	65(10.30%)	62.02(9.83%)	4(5.63%)	6.98(9.83%)			
Emergency Cesarean Section	92(14.58%)	108.76(17.25%)	29(40.85%)	12.24(17.25%)			
Anesthesia					**6.84**	**1**	**< .01**
No	303(48.10%)	292.08(46.36%)	22(30.99%)	32.92(46.36%)			
Yes	327(51.90%)	337.92(53.64%)	49(69.01%)	38.08(53.64%)			

Episiotomy					.78	1	.376
No	514(83.31%)	510.87(82.80%)	54(78.26%)	57.13(82.80%)			
Yes	103(16.69%)	106.13(17.20%)	15(21.74%)	11.87(17.20%)			
Complications during Delivery					**6.19**	**1**	**.013**
No	567(91.45%)	560.70(90.43%)	57(81.43%)	63.30(90.43%)			
Yes	53(8.55%)	59.30(9.57%)	13(18.57%)	6.70(9.57%)			
Person of Trust					**5.56**	**1**	**.018**
No	146(23.14%)	154.60(24.50%)	26(36.63%)	17.39(24.50%)			
Yes	485(76.86%)	476.40(75.50%)	45(63.38%)	53.60(75.50%)			
Full Term					.55	1	.457
No	50(7.92%)	52.13(8.26%)	8(11.27%)	5.87(8.26%)			
Yes	581(92.08%)	578.87(91.74%)	63(88.73%)	65.13(91.74%)			
Infant's Health Complications					.01	1	.976
No	459(78.33%)	459.59(78.43%)	50(79.37%)	49.41(78.43%)			
Yes	127(21.67%)	126.41(21.57%)	13(20.63%)	13.59(21.57%)			
**Post-partum related Factors**							
Information Infant's Health					**41.34**	**1**	**< .001**
No	88(13.97%)	107.85(17.12%)	32(45.07%)	12.15(17.12%)			
Yes	542(86.03%)	522.14(82.88%)	39(54.93%)	58.85(82.88%)			
Rooming-in					**12.59**	**1**	**< .001**
No	170(26.94%)	183.37(29.06%)	34(47.89%)	20.63(29.06%)			
Yes	461(73.06%)	447.63(70.94%)	37(52.11%)	50.36(70.94%)			

Note. Low-risk mothers are those who scored below the 90th percentile cut-off score of 25, while high-risk mothers are those who scored at or above the 90th percentile cut-off score of 25.

3. The Comparison between Different Versions of the PPQ-II

Table S4.

Comparison of the Italian PPQ-II Validation and Previous Versions.

Version	Factors	Items	Representing	Variance proportion
English	F1	7,8,10,12	Arousal	44%
	F2	2,4,5,14	Avoidance	13%
	F3	1,3,13	Intrusion	8%
Korean	F1	7,8,9,10,11,12,13	Arousal	44.7%
	F2	4,5,6	Avoidance	7.2%
	F3	1,2,3,14	Intrusion	15.1%
Chinese	F1	7,8,9,10,11,12,13	Arousal	32%
	F2	4,5,14	Avoidance	11%
	F3	1,2,3	Intrusion	8%
Turkish	F1	1,2,3,4,5,13	Intrusive Thoughts and Avoidance,	12%
	F2	6,7,8,9,10,11,12	Hyperarousal and Numbness Responses	43.60%
Spanish	F1	7,8,9,10,11,12,13	Arousal	43%
	F2	2,4,5,6,14	Avoidance	13.8%
	F3	1,3	Intrusion	6.3%
Portugese	F1	7,8,9,10,11,12,13	Arousal	30.3%
	F2	1,2,4,5	Intrusion and Avoidance	18%
Italian	F1	7,8,9,10,11,12	Arousal and Mood Alteration	26%
	F2	1,2,4,5	Avoidance and Intrusion	21%

4. The validation process of the Italian version of PPQ-II

Figure S1.

Flowchart of the Italian PPQ-II validation process.

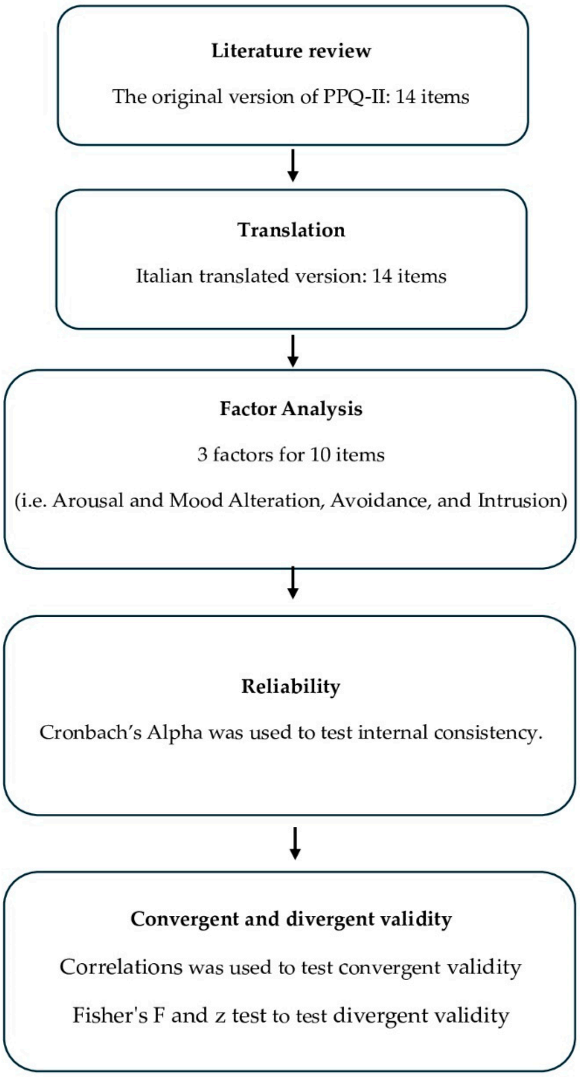

Used R packages

R. R Core Team (2020). R: A language and environment for statistical computing. R Foundation for Statistical Computing, Vienna, Austria.
https://www.R project.org/.

car. Fox J, Weisberg S (2019). An R Companion to Applied Regression, Third edition. Sage, Thousand Oaks CA.
https://socialsciences.mcmaster.ca/jfox/Books/Companion/

cocor. Diedenhofen, B. & Musch, J. (2015). cocor: A Comprehensive Solution for the Statistical Comparison of Correlations. PLoS ONE, 10(4): e0121945.
http://dx.doi.org/10.1371/journal.pone.0121945

GPArotation. Bernaards CA, Jennrich RI (2005). Gradient Projection Algorithms and Software for Arbitrary Rotation Criteria in Factor Analysis. Educational and Psychological Measurement, 65, 676-696.
https://doi.org/10.1177/0013164404272507

parameters. Lüdecke D, Ben-Shachar M, Patil I, Makowski D (2020). Extracting, Computing and Exploring the Parameters of Statistical Models using R. Journal of Open Source Software, 5(53), 2445.
https://doi.org/10.21105/joss.02445
